# Impact of Income, Density, and Population Size on PM_2.5_ Pollutions: A Scaling Analysis of 254 Large Cities in Six Developed Countries

**DOI:** 10.3390/ijerph18179019

**Published:** 2021-08-26

**Authors:** Moon-Jung Kim, Yu-Sang Chang, Su-Min Kim

**Affiliations:** 1 Department of Business, Gachon University, 1342 Seongnam-daero, Sujung-gu, Seongnam 13120, Gyeonggi-do, Korea; mjkim61@gachon.ac.kr; 2 Gachon Center for Convergence Research, Gachon University, 1342 Seongnam-daero, Sujung-gu, Seongnam 13120, Gyeonggi-do, Korea; ysc999@gachon.ac.kr

**Keywords:** PM_2.5_ concentrations, city income per capita, population density, population size, STIRPAT model, threshold regression, environmental Kuznet curve

## Abstract

Despite numerous studies on multiple socio-economic factors influencing urban PM_2.5_ pollution in China, only a few comparable studies have focused on developed countries. We analyzed the impact of three major socio-economic factors (i.e., income per capita, population density, and population size of a city) on PM_2.5_ concentrations for 254 cities from six developed countries. We used the Stochastic Impacts by Regression on Population, Affluence and Technology (STIRPAT) model with three separate data sets covering the period of 2001 to 2013. Each data set of 254 cities were further categorized into five subgroups of cities ranked by variable levels of income, density, and population. The results from the multivariate panel regression revealed a wide variation of coefficients. The most consistent results came from the six income coefficients, all of which met the statistical test of significance. All income coefficients except one carried negative signs, supporting the applicability of the environmental Kuznet curve. In contrast, the five density coefficients produced statistically significant positive signs, supporting the results from previous studies. However, we discovered an interesting U-shaped distribution of density coefficients across the six subgroups of cities, which may be unique to developed countries with urban pollution. The results from the population coefficients were not conclusive, which is similar to the results of previous studies. Implications from the results of this study for urban and national policy makers are discussed.

## 1. Introduction

Heavy fine particulate (PM_2.5_) pollution has increased and become a high risk to public health in densely populated urban areas in many countries. According to a recent study involving 381 large cities with populations of more than 0.75 million people in China, India, the U.S., Europe, Latin America, and Africa [1], the annual average PM_2.5_ concentrations from 2000 to 2006 in 23.9% of these cities was higher than the World Health Organization’s (WHO) interim Target 1 of less than 35 micrograms per cubic meter (35 μg/m^3^). In addition, only 18.0% of these large cities were within the recommended WHO target of less than 10 μg/m^3^. Large cities in Asia, especially China and India, had the worst record, with 48.7% of these cities recording PM_2.5_ concentrations higher than 35 μg/m^3^ and only 1.7% with PM_2.5_ concentrations of less than 10 μg/m^3^. In contrast, large cities in Latin America had the best air quality, with 64.4% of them within the 10 μg/m^3^ guideline.

Translating urban pollution risk in terms of the number of people, it has been reported [1] that more than 500 million Chinese urban residents (14% of the global urban population) were at risk from PM_2.5_ hazard (35 μg/m^3^ or more) in 2010. These people resided in 154 cities, which represented 78% of all large cities with a population of more than 1 million. To make matters worse, 278 million more people became exposed to PM_2.5_ hazards between 2000 and 2010 due to the high birth rate and high migration rate from rural areas to these cities.

In short, the air pollution risk appears to be far more serious in urban settings in large cities in several countries in Asia, particularly in China, India, and Pakistan. Therefore, a high priority for research on socio-economic influencing factors of PM_2.5_ pollution in urban centers is needed to develop effective mitigating policies to control urban pollution. However, the unavailability of relevant city-wide technology-related data, such as the industrial structure, energy intensity, service structure, vehicle usage, as well as income per capita has been a barrier to the productive flow of research. Fortunately, however, the unavailability of city-wide data has become somewhat more manageable in recent years, increasing the number of necessary studies.

A large majority of these studies have focused exclusively on urban pollution in China [2,3,4,5,6,7,8,9,10,11,12]. For example, Hao and Liu [2] examined the four influencing factors of GDP per capita, industrial structure, vehicle population, and population density to PM_2.5_ concentrations for 73 Chinese cities. The results showed that secondary industries, including manufacturing, construction, fast moving consumer goods, and other industries, and the vehicle population, in that order, had greater impacts on PM_2.5_ concentrations in these cities. Wu et al. [3] used PM_2.5_ data from the same 73 cities in 2013 and 2014 and determined that PM_2.5_ significantly correlated with the proportion of industrial activity, the number of vehicles, and household gas consumption in these cities.

Expanding the number of cities to 338 Chinese cities from 2014 to 2017, Wang et al. [5] determined that population density and the number of vehicles had a large impact on increasing PM_2.5_, and GDP per capita had a moderate impact on PM_2.5_. Cheng et al. [4] also used STIRPAT models to analyze influencing factors of PM_2.5_ concentrations for 285 Chinese cities from 2001 to 2012. Their results indicated that population density, income, and traffic intensity had a significant impact on PM_2.5_ concentrations. In addition, secondary industries and central heating significantly aggravated urban air pollution. However, foreign direct investment was not a significant factor.

In contrast to the many studies on urban pollution, only a few socio-economic studies on PM_2.5_ concentrations in cities in developed countries have been published in recent years [13,14]. The current study fills this important gap in the urban pollution literature for developed economies by focusing on 254 cities in the U.S., Germany, Japan, France, U.K. and Spain. More specifically, a STIRPAT framework was used to analyze the three influencing factors of population, income, and technology on PM_2.5_ concentrations from 2001 to 2013.

After this introduction, the paper has four sections. A brief literature review on selected socio-economic studies on PM_2.5_ pollution is presented in the next section, followed by a section explaining the STIRPAT model and data sources. An analysis of the results is presented in the fourth section. Finally, the conclusion, implications, and limitations of the study are presented in the fifth section.

## 2. Literature Review of PM_2.5_ Concentrations in Urban Centers

The large majority of socio-economic studies on PM_2.5_ concentration in urban centers in recent years have concentrated on China [2,3,4,5,6,8,9,10,12,15]. One reason is that PM_2.5_ is the main component of haze and fog for large cities in China, so investigating the relationship between socio-economic factors related to PM2.5 pollution is very important to Chinese policy makers. Another reason is that city-level data for PM2.5 pollutants and related socio-economic factors in many other developing countries are mostly unavailable. In terms of developed countries, PM_2.5_ is not as critical of an environmental pollution issue for cities in developed countries [2]. Thus, only a few socio-economic studies on PM_2.5_ concentrations for cities in developed countries have been published recently on cities in the U.S. [13] and Germany [14]. A group of related studies on air pollution in developed countries such as the U.S. and Canada have also been published [16,17,18,19,20,21]. A few other papers on multiple cities in both developed and developing countries have also been published [1,22,23].

We selected the five most representative papers on the socio-economic analysis of city-level PM_2.5_ concentrations in China for a closer examination [2,4,8,9,15]. The results show that economic development measured by city GDP per capita and population density are the two most influential factors analyzed by these five studies, followed by traffic intensity analyzed by four articles, and industrial structure and energy or electricity intensity examined by three articles. Other factors such central heating, trade openness, and foreign direct investment were analyzed in one article each.

The most consistent finding is related to the increasing or decreasing impact of city GDP per capita to PM_2.5_ concentrations, depending on the income level of cities. All five articles found a statistically significant inverse U-shaped or inverted N-shaped environmental Kuznet curve (EKC). For example, Cheng et al. [4] estimated that 83.4% of Chinese cities are below the inflection point of EKC, and as a result of increasing income, have experienced an increase in PM_2.5_ pollution. Similarly, Liu et al. [9] concluded that high- income cities in China have surpassed the peak of their EKC while upper- and low-middle income cites have not.

Wu et al. [8] also estimated that most cities in the eastern region with higher incomes have passed the inflection point, while cities in the middle region may need 10 to 15 years to reach their peak of EKC. To elaborate, Wu et al. [8] verified the existence of an inverted U-shaped EKC involving 104 cities in the middle region of China with the inflection point estimated at $18,506 per capita. As of 2011, only 11% (13) of these cities have arrived at this inflection point, creating a win-win relationship between income and PM_2.5_ pollution. For 108 cities in the eastern region, there is an inverted N-shaped curve with a projected inflection point of $9186. As many as 64% (69) of the cities have reached their inflection point, and another 18% (19) cities are expected to reach their inflection point within the next five years. The remaining 47 cities in the western region do not follow the EKC and show a linear positive relationship between income per capita and PM_2.5_ pollution.

Wang and Fang’s [6] study on 53 cities in the Bohai Rim Urban Agglomeration found that 43 of the 53 cities displayed a negative relationship with GDP per capita with an average coefficient of −1.8. In other words, an increase of 10,000-yuan GDP per capita would cause a reduction of 1.18% of μg/m^3^ in PM_2.5_.

Nearly the same findings can be found for industrial structure, measured by the proportion of value added by secondary industry to GDP as well as traffic intensity measured by the proportion of the number of civilian vehicles to the total length of urban roads. In short, four of the five articles (with the exception of Wu et al. [8]) confirmed a positive and significant relationship between high traffic intensity and high secondary industry output to higher PM_2.5_ concentrations.

The impact of population density on PM_2.5_ pollution has had somewhat contradictory results in these five articles. The findings by Cheng et al. [4], Wu et al. [8], and Zhou et al. [15] were statistically significant and positive. For example, Cheng et al. [4] showed that population density coefficients to PM_2.5_ concentrations derived from three separate panel regression models for 285 cities in China from 2001 to 2012 generated all six population density coefficients ranging from +0.06 to +0.029, which were statistically significant at the 1% level. In other words, a 1% increase in density increased PM_2.5_ concentrations from 0.029% to 0.06%, while the effects from other factors such as income, industrial structure, electricity intensity, traffic intensity, and several others held constant. The other two articles by Hao and Liu [2] and Lin et al. [24] showed positive but no statistically significant density coefficients.

Another factor, energy or electricity intensity, has also generated somewhat contradictory findings in the three articles. For example, Cheng et al. [4] found a statistically significant positive impact of increased electricity consumption on increased PM_2.5_ pollution. Similarly, Wu et al. [8] found a strong positive impact of coal consumption on PM_2.5_ pollution. In contrast, Zhou et al. [15] found no significant relationship between electricity consumption and PM_2.5_ pollution.

It is interesting to note that none of the five articles examined the impact of the population size of cities on PM_2.5_ pollution. However, other papers such as Wang et al. [25] reported a positive correlation between PM_2.5_ concentrations and urban population, together with the size of the urban areas, the share of secondary industry, and population density. Han et al. [11] and Han et al. [26] suggested that urbanization had a considerable impact on increasing PM_2.5_ concentrations in Chinese cities.

The findings from socio-economic analyses of urban PM_2.5_ pollution in developed countries are less clear as they vary from those reported on city level PM_2.5_ concentrations in China. A recent socio-economic analysis of PM_2.5_ pollution on cites in developed countries emphasized the role of population density over income or population size. For example, Carozzi and Roth [13] found a positive and statistically significant population density coefficient of +0.13 for PM_2.5_ concentrations. Specifically, they found that doubling the density would increase the average PM_2.5_ pollution roughly 10% across 933 U.S. cities.

In another systematic study on 109 districts in Germany, which included 51 urban districts, Borck and Schrauth [14] found that a 1% increase in population density increased PM_2.5_ concentrations by a modest 0.08%. Using an authoritative survey, Ahlfeldt and Pietrostefani [27] cited both studies by Carozzi and Roth [13] and Borck and Schrauth [14], and recommended +0.13 as the elasticity for pollution reduction. In short, the impact of high-density cities on PM_2.5_ pollution was positive in Chinese studies. Similarly, studies on U.S. and Germany cities also suggested that the effect of high population density was moderately positive.

However, when population density is examined in the framework of urban spatial structure or urban form in relation to air pollution, studies of American and European cities have shown that low-density urban sprawl can lead to a significant deterioration of air quality [16,19,28,29].

For example, Bereitsnaft and Debbaze [19] found that among 86 metropolitan areas in the U.S., low-density urban sprawls led to higher concentration of air pollution. Stone [16] showed for the 45 major cities in the U.S, the more compact the city, the smaller the spread, the more likely it was to reduce air pollution emissions. The primary reason is that compact low-density cities can reduce transportation emissions and air pollution, due to the proximity of housing and employment. In contrast, Clark et al. [30] found that PM_2.5_ pollution levels increase as population density increases. Another study for 249 European cities found that high density cities were more vulnerable to high levels of SO_2_ concentration [31].

In sum, it is likely that both very high- and very low-density cities may be subjected to higher levels of air pollution emissions. Thus, several recent studies have proposed adjusting population density upward by promoting monocentric urban structures for those low-density cities while adjusting population density downwards by promoting polycentric structures for the excessively high-density cities as possible remedies to reduce air pollution [32,33,34].

As for the impact of income on PM_2.5_ pollution, Anenberg et al. [22] discovered that PM_2.5_ concentrations across 82 global cities were negatively associated with city GDP per capita at a correlation coefficient of 0.64 at *p* < 0.0001. In other words, the negative impact from higher income cities in developed countries on PM_2.5_ pollution appears to be more pervasive compared to Chinese cities.

Another paper by Ouyang et al. [35] examined the driving forces of PM_2.5_ concentrations in 30 OECD countries from 1998 to 2015 using a threshold panel model. The result was that a 1% increase in GDP per capita decreased the PM_2.5_ concentrations from 0.3% to 0.4%, depending on the three income levels of countries. These findings supported the earlier studies by Wang and Fang [6] and Wang et al. [25].

As for the impact of population size of a city on PM_2.5_ pollution, Han et al. [1] discovered an inverse U-shape relation among Chinese cities. However, they indicated that the relationships in U.S., European, and Latin American cities were stationary or showed a small increasing trend. In other words, the larger population size cities in China may be more likely to experience higher PM_2.5_ pollution than those in U.S., European, and Latin American cities. For cities in India and Africa, they discovered a U-shaped trend for PM_2.5_ concentrations as urban population increased.

## 3. Method and Data

### 3.1. STIRPAT Model

Many scholars have used econometric models to analyze the influencing factors of energy usage and air pollution from a socio-economic perspective. Econometric models include both cross-section and panel models. The use of panel models has become popular as they can increase the sample size, reduce collinearity between variables, and control individual heterogeneity of samples to improve the reliability and validity of the estimates [4].

The original IPAT model was refined later to become the Stochastic Impacts by Regression on Population, Affluence and Technology (STIRPAT) model, which enabled researchers to estimate the proportional change of the environmental impact per given proportional change in population, affluence, and technology.

The STIRPAT model is defined as

(1) $$I_{it}=aP_{it}{}^{b}A_{it}{}^{c}T_{it}{}^{d}e_{it}$$

where I represents the pollution intensity of a pollutant, P represents the total population, A depicts affluence or income, and T indicates the level of technological development. Subscript i and t of each variable denote the cross-sectional unit, which is the cites and time period, respectively; a is the constant; b, c, and d are the exponents of P, A, and T, respectively, to be estimated; and e is the residual error term.

To ease the task of estimating exponents, Equation (1) is converted into the log-log form of Equation (2) by taking the natural log of both sides.

(2) $$InI_{it}=In\left(a_{it}\right)+b\left[In\left(P_{it}\right)]+c[In\left(A_{it}\right)]+d[In(T_{it}\right)]+e_{it}$$

The natural log is helpful as it converts non-linear variables to linear ones, rendering the results interpretable as a percentage change. For example, b can be viewed as the population elasticity that measures the percentage change of the environmental impact resulting from a 1% change in population. The STIRPAT model has also been used to examine the impact of population, income, and/or technology in other areas such as the material footprint, human ecological footprint, and environmental efficiency of well-being [36,37,38].

Many scholars have also used the STIRPAT model to analyze the impact of socio-economic factors on PM_2.5_ pollution at the country level as well as the city level [39,40,41,42,43,44,45,46]. As for a measure for technology, there is no consensus on a single measure of technology [47]. According to Cole and Neumayer [48], technology is a broad term intended to reflect technological, cultural, and institutional determinants of the environmental impact. For example, Uddin, Alam and Gow [49] extensively used the urbanization ratio measured as the percentage of the population living in urban areas for technology in their STIRPAT model. Wang et al. [40] also used the urbanization ratio, together with energy intensity for the technology factor in their STIRPAT model.

For this study, population density is used to represent the technology factor in the STIRPAT model, together with the population size of a city as P and income per capita of a city as A, as shown in Equation (3):

(3) $$InY_{it}=Ina+b\left(InP_{it}\right)+c\left(InA_{it}\right)+d\left(InPD_{it}\right)+e_{it}$$

where P represents the population size of each city, A represents the GDP per capita of a city, and PD represents population density calculated by population per km^2^.

In addition, we use threshold regression as a robust test to verify the results from the STIRPAT analysis. This study uses Hansen’s [50] threshold regression using the simplest form of regression where a single threshold was called for. The single threshold regression model includes Equations (4) and (5):

(4) $$Y_{i}=\theta_{1}x_{i}+e_{i}\;q_{i}\leq \gamma$$

(5) $$Y_{i}=\theta_{2}x_{i}+e_{i}\;q_{i}>\gamma$$

where i represents the units of analysis, which is a city; *Y* represents the dependent variable of PM_2.5_ concentrations; x represents the explaining variables of population size (P), income per capita (I), and population density (PD); *θ*1 and *θ*2 represent parameters to be estimated; *q* represents the threshold variable; γ represents the threshold quantity; and *e* represents the error term. Based on the variables selected in this study, the threshold model is expressed in Equations (6) and (7):

(6) $$InY_{i}=\theta_{1}\left[InP_{i}+InI_{i}+InPD_{i}\right]+e_{i}\;,\;\left(Inq_{i}\leq In\gamma \right)$$

(7) $$InY_{i}=\theta_{2}\left[InP_{i}+InI_{i}+InPD_{i}\right]+e_{i}\;,\;(Inq_{i}>In\gamma )$$

We then combine Equations (6) and (7) using a dummy variable, which takes the value of one when the condition in parentheses is met, otherwise it becomes zero. This combined equation is used as the estimation equation of this research. The generalized threshold panel model has been used extensively in the fields of energy consumption, renewable energy development, and carbon emission on sustainable development [51,52,53,54,55,56,57].

### 3.2. Data and Data Sources

We downloaded the data set indicating the exposure to PM_2.5_ in metropolitan areas from 2001 to 2013 (https://stats.oecd.org/index.aspx?DatasetCode=EXP_PM2_5_FUA accessed on 12 August 2020) for 706 cities in six countries: the U.S. (262 cities), Germany (109 cities), the U.K. (101 cities), France (82 cities), Japan (76 cities), and Spain (76 cities).

We then downloaded the population size, metropolitan land area, and GDP from 2001 to 2013 (http://stats.oecd.org accessed on 24 August 2020). After eliminating cities with missing data, we obtained the final sample size of 254 cities with a complete set of yearly data on PM_2.5_, population size, population density, and city GDP per capita. The final sample size of 254 cities included 59 cities in the U.S., 57 cities in Germany, 46 cities in Japan, 38 cities in France, 33 cities in the U.K., and 21 cities in Spain.

Further details on data and data sources are presented in Table 1. First, the PM_2.5_ mean pollution exposure was 13.15 μg/m^3^ and the median was nearly the same at 13.05 μg/m^3^. Second, the population size of the cites during the study period was calculated at 1.35 million inhabitants. The average city GDP income per capita measured in constant international U.S dollars with a base year of 2010 at PPP was $37,772. Finally, the average population density during the study period was 703.68 persons per km^2^.

For the subgroup analysis, the total sample of 254 cities was independently ranked from highest to the lowest in each of the three categories of income, density, and population size. We used the latest income figures of 2013 to categorize the income subgroups. The total group of 254 cities was categorized into two equal numbers of the top 127 highest income cities and the bottom 127 lowest income cities. The top 127 subgroup was led by San Francisco, CA, the highest ranked for income per capita at $84,921, and ended with Rennes, France, the 127th ranked at $35,966. The bottom 127 subgroup was led by Reims, France, ranked 128th at an income per capita at $35,964, and ended with Cordoba, Spain, ranked 254th at $22,057. To highlight the scale effect, three additional subgroups were created: the top 15, top 30, and top 60 high-income cities. The top 15 subgroup was again led by San Francisco, CA and the 15th ranked Denver, CO at $60,752, while the top 30 subgroup was led by San Francisco, CA and the 30th ranked Aberdeen, SD at $54,812. Finally, the top 60 subgroup was led again by San Francisco, CA and the 60th ranked Sacramento, CA at $44,680.

We then applied the same procedure to population density using 2013 density data. For density subgroups, the top 127 subgroup was led by the first ranked Tokyo, Japan, at 8635 persons per km^2^ and ended with the 127th ranked Hachinohe, Japan at 405 persons per km^2^. The bottom 127 subgroup was led by the 128th ranked Granada, Spain at 402 persons per km^2^ and ended with the 254th ranked Albuquerque, NM at 28 persons per km^2^. To highlight the scale effect, we created three additional subgroups of the top 15, top 30, and top 60 high-population density cities. The top 15 subgroup was again led by Tokyo, Japan and ended with the 15th ranked Barcelona, Spain at 2076 persons per km^2^. The top 30 subgroup was led by Tokyo, Japan, and ended with the 30th ranked Santa Cruz de Tenerife, Spain at 1311 persons per km^2^. Finally, the top 60 subgroup was led again by San Francisco, CA and ended with the 60th ranked Bonn, Germany at 797 persons per km^2^.

For population size subgroups, using 2013 population data, the top 127 subgroup was led by the highest-ranked Tokyo, Japan at 35,221,137 inhabitants and ended with the 127th ranked Toulon, France at 553,594 inhabitants. The bottom 127 subgroup was led by the 128th ranked Numazu, Japan at 553,358 inhabitants and ended with the 254th ranked Tuscaloosa, AL in the USA at 244,054 inhabitants. To highlight the scale effect, we again created three additional subgroups of the top 15, top 30, and top 60 high-population size cities. The top 15 subgroup was again led by Tokyo, Japan and ended with the 15th ranked Berlin, Germany at 4,950,913 inhabitants. The top 30 subgroup was led by Tokyo, Japan and ended with the 30th ranked Sacramento, CA at 2,213,564 inhabitants. Finally, the top 60 subgroup was led again by Tokyo, Japan and ended with the 60th ranked Bremen, Germany at 1,230,691 inhabitants. Detailed ranking of the cities by income, density, and population are listed in the Appendix A Table A1, Table A2 and Table A3.

## 4. Analysis of Results

This study used the panel unit root test to check whether the data used in this study were stationary or not. We applied two widely used tests: the Levin–Lin–Chu (LLC) test developed by Levin, Lin, and James [58], and the Fisher Phillips–Perron (PP) test developed by Phillips and Perron [59]. The results indicated that there is a common unit root process in all of the variables, with one exception of income in the Fisher PP test, as shown in Table 2.

We then tested for multicollinearity among the explanatory independent variables in all of the panel regression models using variance inflation factors (VIFs). The VIF values were all less than 10, as shown in Table 3, suggesting no multicollinearity [60].

In the regression of the SPIRPAT model, this research used the Prais–Winsten (PW) estimation method with panel-corrected standard error. The PW method uses a generalized least square framework that corrects for AR(1) autocorrelation within the panels and cross-sectional correlation and heteroscedasticity across panels [61].

STIRPAT multivariate panel regression of PM_2.5_ concentrations on the three separate groups of 254 cities by income, density, and population, and their respective five subgroups generated the following variable results. First, the full sample of 254 cities ranked by 2013 income per capita yielded a statistically significant −0.074. In other words, a 1% increase in income per capita generated a −0.074% reduction of PM_2.5_ concentrations, while the impact from the other factors of density and population size were held constant, as shown in Table 4.

When the subgroup of 254 cities was divided into the top 127 high-income cities, the income coefficient increased to −0.208, which was about 2.7 times larger than the income coefficient obtained from the 254 cities. A 1% increase in income per capita reduced PM_2.5_ concentrations by 0.208% for the subgroup of 127 high-income cities. In contrast, the sample of the remaining bottom 127 low-income cities yielded a statistically significant coefficient of +0.157, while the other factors held constant. Specifically, a 1% increase in income increased PM_2.5_ concentrations by 0.157%, which indicated an income scale disadvantage for the bottom 127 low-income cities. These results suggest that there are effects from the EKC curve on cities with different income levels.

Furthermore, the contrasting results from the top 127 and the bottom 127 cities suggested the possibility of an even greater income scale advantage for cities with very high income per capita. Therefore, extended STIRPAT analysis of income coefficients for the samples of the top 15, top 30, and top 60 high-income cities were examined. The result was that the top 60 cities yielded −0.505, while the top 30 yielded −0.582. Finally, the top 15 high-income cities generated the highest negative income coefficient of −0.783, which was about 10 times larger than the income coefficient of the 254 cites at −0.074, indicating the existence of a very large-scale advantage of income for PM_2.5_ pollution. Furthermore, all three income coefficients met the statistical test of significance.

In summary, a very large-scale advantage of income for reduced PM_2.5_ pollution was evident in the top 15, top 30, and top 60 high-income cities. This income scale advantage continued through the top 127 cities at a somewhat reduced scale. However, the scale advantage showed a slight scale disadvantage for the remaining bottom 127 cities with lower income per capita. The full sample of 254 cities showed a moderate yet statistically significant income scale advantage by combining different income coefficients from these city subgroups.

The same STIRPAT multivariate panel regression was applied first to the full sample of 254 cities, ranked by 2013 population density. The density coefficient from the full sample of 254 cities yielded statistically significant density coefficients of +0.058, as shown in Table 5, which resembled the results in a German study [14] with a density coefficient of +0.08. In other words, a 1% increase in density yielded a 0.058% increase in PM_2.5_ concentration, demonstrating that the impact of density on PM_2.5_ concentrations was positive. When the full sample of 254 cities was divided into the subsample of the top 127 cities with higher density, the density coefficient was more or less unchanged at 0.053, meeting the statistical test of significance, while the effects from the other factors held constant.

The bottom 127 cities with lower densities yielded a statistically significant density coefficient of +0.142, which was substantially higher than the density coefficients of both the top 127 and all 254 cities. In other words, the positive impact of population density on PM_2.5_ pollution was much greater for the group of cities with lower densities compared to the group of cities with higher densities.

The result for the top 60 cities yielded a statistically significant coefficient of +0.05, which was nearly the same as the +0.058 coefficient estimated for all 254 cities. Similarly, the density coefficients for the top 30 cities remained at +0.061. However, for the top 15 cities, the density coefficient more than doubled to +0.13. The density coefficients for the top 15 cities did not meet the statistical test of significance, whereas the density coefficient for the top 30 cities did.

In summary, all of the density coefficients displayed a positive impact of density on greater PM_2.5_ pollution. The positive impact was greater for the 127 cities with lower population densities over both the 127 cities with higher population densities, and the full sample of all 254 cites. The top 60 and 30 cities displayed density coefficients nearly equal to those of the top 127 cities and all 254 cities. However, the top 15 cities displayed a substantially higher density coefficient, approaching the density coefficient derived from the bottom 127 cities. In sum, the overall pattern of density coefficients followed a U-shaped pattern, providing an interesting contrast to the inverse U-shaped pattern of the EKC.

Finally, the full sample of 254 cities ranked by 2013 population size was subjected to the same panel regression analysis. The resulting population coefficient of −0.018 met the statistical test of significance. To explain, a 1% increase in population would reduce PM_2.5_ concentrations slightly by 0.018% for the full sample of 254 cities, while the effects of the other factors of income and density were held constant, as listed in Table 6. However, compared to the income and density coefficients estimated earlier, the magnitude of impact of population size is quite moderate. In addition, similar to the effect of income, larger population sizes implied a smaller reduction in PM_2.5_ concentrations.

To differentiate the degree of impact between large versus small population size, the full sample of 254 cities was again divided into the subgroups of the top 127 largest population cities and the remaining 127 smallest population cities. The resulting population coefficient for the top 127 cities was much smaller at −0.009, compared to the −0.018 estimated for all 254 cities, but the coefficient failed to meet the statistical test of significance. The remaining 127 cities with smaller populations yielded a substantially larger population coefficient of +0.024, which again did not meet the statistical test of significance.

To determine the impact of population mega cities of the top 15 most populated cities, the panel regression yielded a statistically significant population coefficient of +0.261. For the subgroup of the top 30 most populated cities, the population coefficient was substantially smaller at +0.027, but failed to meet the statistical test of significance. For the top 60 cites, the population coefficient yielded −0.034, indicating the same population impact on PM_2.5_ concentrations as the subgroups of the top 127 and all 254 cities. However, only the two population coefficients for the top 15 cities and all 254 cities met the statistical test of significance.

In sum, although an increasing population size for the full sample of 254 cities yielded a moderate reduction in PM_2.5_ pollution, the results from the subgroup analyses did not support such an impact. On the contrary, the subgroup of 15 mega cities indicated a much higher impact of increasing, not decreasing, PM_2.5_ pollution. The results from the remaining subgroups were inconclusive.

In order to verify the appropriateness of subgroups used in this study so far, the multivariate panel regression was replicated for the four additional subgroups for the respective independent variables. Specifically, we added the subgroups of the top 20, top 50, top 100, and top 200 cities. Table 7 shows the newly derived income, density, and population coefficients for the newly added four subgroups together with the coefficients estimated earlier for the five subgroups of the top 15, top 30, top 60, top 127, and bottom 127 cities.

The four newly estimated income coefficients, all of which are statistically significant, follow the overall declining pattern of coefficients from the top 15 to bottom 127 cities in perfect alignment, indicating the robustness of our previous estimation of the five subgroups. As for the four new density coefficients, they, in general, also support the overall “U” shaped pattern, with a wide flat bottom displayed by the previous five coefficients. The distribution of the four new population coefficients, also, support the overall pattern established by the previously estimated coefficients, where the top 15 displayed the highest coefficients. Similarly, the new coefficients from the subgroup of top 20 cities also displayed the highest coefficients among the new four subgroups.

Since both the top 127 largest cities and the bottom 127 smallest cities failed to generate statistically significant population coefficients, we used an alternative model of threshold regression as a robustness check. The results, shown in Table 8, indicated that the optimal single-threshold value was estimated at 0.768 million inhabitants. We divided the full sample of 254 cities into Region 1 with more than 0.768 million inhabitants and Region 2 with less than 0.768 million inhabitants. Region 1 with 94 cities had an average population size of 2,903,502 inhabitants and Region 2 with 160 cities had an averaged population size of 436,966 inhabitants.

The population coefficient for Region 1 generated a statistically significant +0.045, compared to −0.009 for the top 127 cities, whereas Region 2 generated a statistically insignificant +0.012, compared to +0.024 for the bottom 127 cities.

In sum, the robustness test with threshold regression using the subgroups of alternative population size for the top 94 cities and the bottom 160 cities improved the statistical validity for the subgroup of cities with large populations. However, the basic piecewise linear pattern of population coefficients remained essentially intact.

## 5. Conclusions

The key findings from this research can be summarized as follows. First, the impact of income measured by city GDP per capita on PM_2.5_ pollution for the full sample of 254 cities was highest, in that a 1% increase in income generated a −0.074 reduction of PM_2.5_ concentrations. In contrast, the impact of population density was nearly as high, in that a 1% increase in population density resulted in a 0.058% increase of PM_2.5_ concentrations. The impact from population size was quite modest, in that a 1% increase of population size resulted in a reduction of only 0.018%.

Second, when all 254 cities were categorized into five subgroups of the top 127, bottom 127, top 60, top 30, and top 15 cities, the impact of income, density, and population varied so widely that each influencing factor needed a separate in-depth analysis. We provide a summary in Table 9 of the six coefficients for each of the three influencing factors of income, density, and population. We also present the average values during the study period of income, density, and population for all 254 cities as well as for each of the respective five subgroups under analysis.

**Figure 1:**
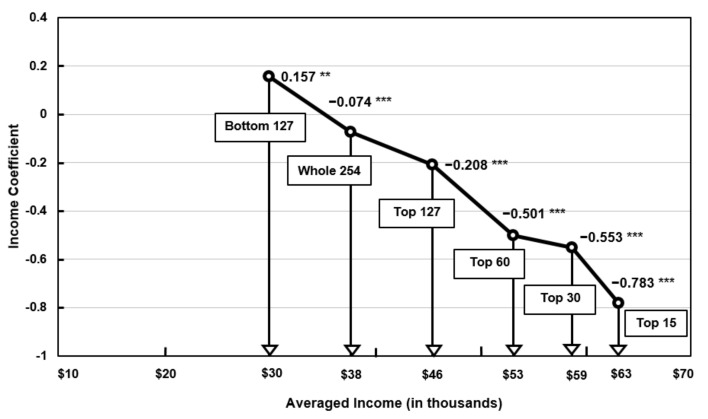
Distribution of income coefficients and averaged income for all cities and the five subgroups of cities (2001–2013). *** *p* < 0.01, ** *p* < 0.05.

Third, the results of the income subgroup analysis showed the most consistent pattern following the EKC. The richest cities displayed the highest scale advantage for greater pollution reduction, whereas the lower income cities experienced a scale of diseconomy with pollution increases. To elaborate, Figure 1 shows that the income coefficient for the top 15 highest-income subgroup with an average income of $63,132 experienced a reduction of −0.783% in PM_2.5_ pollution, whereas the bottom 127 lower-income cities with an average income of $30,007 experienced a pollution increase of 0.157% for the same 1% increase in income.

To explain this pattern in the context of the EKC, many cities in the subgroup of cities with an average income of $30,007 had not reached the peak of their EKC, and thus, experienced increasing pollution as their income increased. In contrast, many cities in the subgroup with an average income of $45,537 had surpassed the peak, so experienced a win-win relation of increased income and reduced pollution. Cities with the highest income level experienced proportionately greater pollution reductions, as predicted by the EKC.

**Figure 2:**
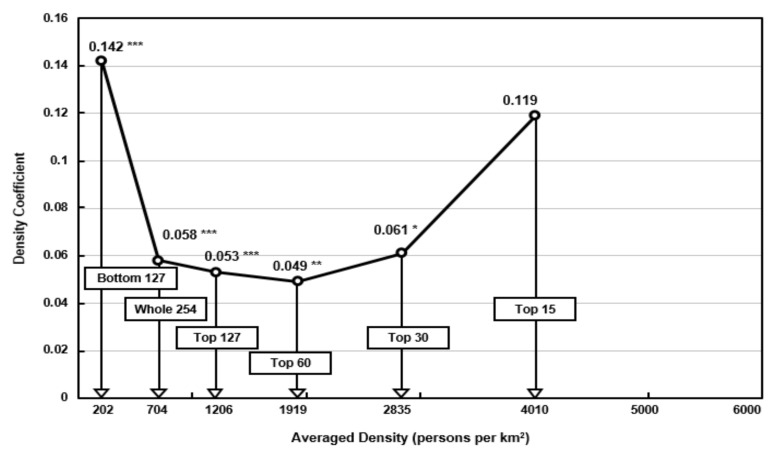
Distribution of density coefficients and averaged density for all cities and the five subgroups of cities (2001–2013). *** *p* < 0.01, ** *p* < 0.05, * *p* < 0.1.

Fourth, the results of the density subgroup analysis showed a somewhat opposite pattern from the income subgroups. As shown in Figure 2, the bottom 127 low-density cites with an average density of 202 persons per km^2^ generated the highest density coefficient of +0.142, whereas the top 15 high-density cities also generated an equally high density coefficient of +0.119. In the remaining subgroups, the density coefficient clustered closely around the density coefficient derived from all 254 cities. Thus, the overall distribution of density coefficients resembled a U-shaped pattern, which is opposite to the inverse U-shaped EKC.

As noted in the earlier section of the literature review, many cities in the U.S and some European countries with low-density urban sprawl may have been responsible for the unusual high-density coefficient of +0.142 estimated for the subgroups of the bottom 127 cities. For example, the bottom 127 subgroup contained a large minority of 36 American cities. Furthermore, this subgroup contained 14 American cities in the bottom 20 lowest density cities, indicating the impact of low-density sprawl cities.

The high-density coefficient of 0.119 from the subgroup of the top 15 cities with a very high average density of 4010 inhabitants per km^2^ may reflect the fact that extremely high-density cities will begin to experience excessive spatial concentration and consequently increasing vehicle emissions due to severe congestion as well as the high number of people exposed to pollution. These can bring about a rapidly rising air pollution. Furthermore, the fact that there are seven high-density Japanese cities included in the top 15 subgroup may have generated another cause for the unusually high coefficient derived for this subgroup.

**Figure 3:**
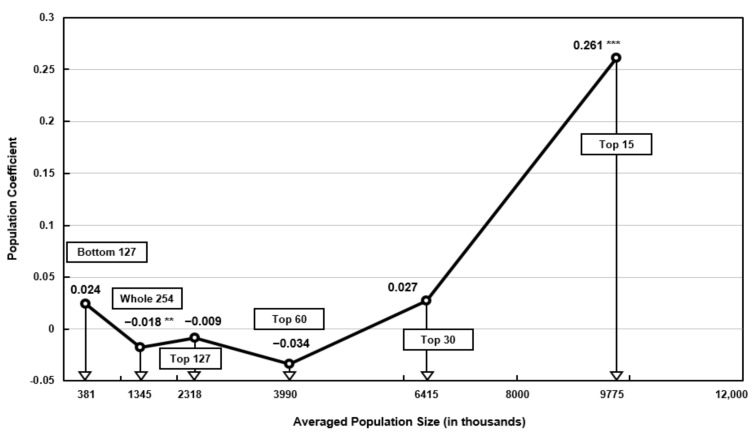
Distribution of population size coefficients and averaged population size for all cities and the five subgroups of cities (2001–2013). *** *p* < 0.01, ** *p* < 0.05.

Fifth, the results from the population subgroups were somewhat inconsistent and contradictory in that only the full sample of 254 cities and the top 15 most populous mega cities generated statistically significant population coefficients. All 254 cities generated −0.018, while the top 15 subgroup generated +0.261. In other words, most cities would experience a very modest pollution reduction in the full sample of 254 cities, whereas the most populous cities in the top 15 subgroup would experience the largest increase in PM_2.5_ pollution. The population coefficient from the remaining subgroups clustered around the population density of the full sample of all cities group. Therefore, the distribution of population coefficients can be approximated using a piecewise linear relation, as displayed in Figure 3. In short, unlike the case of income and density, the population size of cities appears to not have a substantial impact on PM_2.5_ pollution. The only exception was in the case of the most populous 15 mega cities with an average population size around 10 million inhabitants.

It would be interesting to compare the results of this study to the results of studies on Chinese cities discussed earlier in the literature review section. First, the impact of income on PM_2.5_ pollution between the two groups was quite similar, as both the previous studies and our study verified the theory of EKC. One difference may be that the inflection point of the EKC in China could be somewhat lower in the range of $9186 to $18,506 per capita [8]. In comparison, the average income for the bottom 127 cities in this study, generating a positive income coefficient, was estimated at $30,007. Another difference may relate to the very large-scale economy estimated for the top 15 high-income subgroup in this study, which may be different in high-income Chinese cities.

As for population density, both groups of studies verified the result of increasing pollution as a function of increasing population density. However, the U-shaped distribution of density coefficients revealed in this study may be due to many low-density urban sprawls found particularly in the U.S. For example, the average density for all 254 cities was quite low (i.e., estimated at 704 persons per km^2^). In comparison, the average population density for 285 cities in China during a similar study period of 2001 to 2012 was estimated at a much higher density of 1149.86 persons per km^2^ [4]. Finally, both groups of studies found that the impact of population size on PM_2.5_ pollution was inconclusive, although our study revealed rapidly increasing pollution from the most populous cities with an average of 10 million inhabitants.

The findings of this study have policy implications for all countries. An ideal combination of the three influencing factors examined in this study that are most favorable to pollution reduction are (1) an average income per capita of $38,000 or more; (2) population density in the range of 1000 to 2000 population per km^2^; and (3) a medium population size between 1.5 million to 4 million inhabitants. In contrast, the worst combination of the three factors are (1) low-income cities with significantly less than $30,000 per capita; (2) the highest population density of more than 4000 persons per km^2^; and (3) the largest population size of more than 10 million inhabitants.

We realize, however, that such an ideal combination would be quite difficult to achieve in most cities. Fortunately, the results of this study have identified a rather wide indifference zone of the average values in all three factors. For the income, any city income per capita over $38,000 would generate a substantial pollution reduction. For density, the wide indifference zone ranges from about 700 to 3000 persons per km^2^, while the indifference zone of population size ranges from 1.3 million to 6.4 million inhabitants.

There are several limitations to this study that represent possible topics for future studies. One major limitation is the omission of several socio-economic factors related to PM_2.5_ pollution that have been analyzed in previous studies. For example, several previous studies on Chinese cities have included other influencing factors such as industrial structure, traffic intensity, energy and electricity usage, and coal consumption [2,3,4,7]. Another group of omitted factors include meteorological elements such as temperature, precipitation, wind, and humidity [9]. Other omitted variables may include atmospheric chemistry and the long-distance transport of pollution [9,62,63,64,65,66]. These omitted variables could be included in future studies, and thus could revise the interactions related to socio-economic factors examined in this study. In other words, we have provided some evidence for the robust association between the factors of income, density, and population to PM_2.5_ pollutions, rather than evidence of causality. Thus, future work should continue to establish the causal relationships to control air pollutions.

Despite these limitations, this research revealed the role of high-income cities in developed countries and added insights about how pollution reduction can have a greater impact compared to developing countries such as China. This study also supports the positive impact of high population density cities on increasing pollution, which is also the case in developing countries. Going beyond this basic notion, this study proposes a U-shaped pattern of density coefficients as a function of variable population densities of cities for developed countries.

## Figures and Tables

**Table 1:** Data sources for 254 cities.

Variable	Definition	Unit of Measurement	Data Source	Mean	Std.dev.	Min	Max
PM_2.5_	PM_2.5_	Micrograms per cubic meter (μg/m^3^)	OECD statics: Metropolitan areas Environment	13.15	2.83	5	24
POP	Population	No. of population	US Census Bureau (2013)	1,349,779	2,883,295	202,891	35,221,137
ICM	Income	GDP per capita, PPP (constant 2010 international $)	OECD statics: Metropolitan areas Economic	37,772.01	10,616.28	17,367	86,268
DEN	Density	No. of population per km^2^	OECD statics: Metropolitan area Density	703.68	1018.15	20	8635

**Table 2:** Results of panel unit root tests.

Variable	Unit Root Test	
	Levin-Lin-Chu (LLC)	Fisher-PP
lnPM_2.5_	−32.5583 ***	33.1823 ***
lnIncome	−19.5545 ***	−1.7108
lnDensity	−39.4000 ***	14.3177 ***
lnPopulation	−83.3723 ***	68.9691 ***

*** *p* < 0.01.

**Table 3:** VIF test for PM_2.5_ data.

Variable	All	Income					Density					Population				
		Top 15	Top 30	Top 60	Top 127	Bottom 127	Top 15	Top 30	Top 60	Top 127	Bottom 127	Top 15	Top 30	Top 60	Top 127	Bottom 127
lnPopulation	1.64	1.33	1.33	1.47	1.38	1.07	1.31	1.18	1.28	1.43	1.04	2.03	1.61	1.64	1.72	1.05
lnDensity	1.28	1.39	1.69	1.22	1.26	1.35	2.41	2.2	2.28	1.87	1.52	1.08	1.23	1.37	1.74	1.04
lnIncome	1.84	1.21	1.63	1.59	1.68	1.34	2.05	2.31	2.38	2.17	1.53	2.12	1.57	1.48	1.31	1.07
Mean_VIF	1.59	1.31	1.55	1.43	1.44	1.25	1.92	1.89	1.98	1.82	1.36	1.74	1.47	1.5	1.59	1.05

**Table 4:** Multivariate panel analysis of PM_2.5_ concentrations for six income subgroups (2001–2013).

	Subgroups	Top 15	Top 30	Top 60	Top 127	Bottom 127	All 254
Variables							
InIncome		−0.783 ***	−0.553 ***	−0.501 ***	−0.208 ***	0.157 **	−0.074 ***
		(0.225)	(0.122)	(0.080)	(0.043)	(0.068)	(0.028)
InDensity		0.142 ***	0.130 ***	0.121 ***	0.083 ***	0.035 ***	0.058 ***
		(0.033)	(0.023)	(0.013)	(0.009)	(0.009)	(0.007)
InPopulation		−0.011	−0.017	−0.013	−0.033 ***	0.040 **	−0.018 **
		(0.033)	(0.021)	(0.013)	(0.009)	(0.016)	(0.008)
Incons		10.360 ***	8.003 ***	7.434 ***	4.725 ***	0.206	3.225 ***
		(2.470)	(1.307)	(0.792)	(0.411)	(0.692)	(0.252)
R^2^		0.864	0.852	0.848	0.838	0.773	0.812
Observation		195	390	780	1651	1651	3302

*** *p* < 0.01, **p < 0.05.

**Table 5:** Multivariate panel analysis of PM_2.5_ concentrations for six density subgroups (2001–2013).

	Subgroups	Top 15	Top 30	Top 60	Top 127	Bottom 127	All 254
Variables							
InDensity		0.119	0.061 *	0.049 **	0.053 ***	0.142 ***	0.058 ***
		(0.076)	(0.037)	(0.021)	(0.014)	(0.013)	(0.007)
InIncome		0.009	−0.023	−0.025	0.004	−0.171 ***	−0.074 ***
		(0.027)	(0.020)	(0.055)	(0.036)	(0.040)	(0.028)
InPopulation		−0.088	−0.003	0.000	0.001	−0.051 ***	−0.018 **
		(0.148)	(0.092)	(0.014)	(0.010)	(0.012)	(0.008)
Incons		2.416	2.503 ***	2.511 ***	2.161 ***	4.258 ***	3.225 ***
		(1.699)	(0.896)	(0.525)	(0.345)	(0.363)	(0.252)
R^2^		0.824	0.799	0.802	0.810	0.809	0.812
Observation		195	390	780	1651	1651	3302

*** *p* < 0.01, **p < 0.05, * *p* < 0.1.

**Table 6:** Multivariate panel analysis of PM_2.5_ concentrations for six population subgroups (2001–2013).

	Subgroups	Top 15	Top 30	Top 60	Top 127	Bottom 127	All 254
Variables							
InPopulation		0.261 ***	0.027	−0.034	−0.009	0.024	−0.018 **
		(0.072)	(0.036)	(0.021)	(0.013)	(0.029)	(0.008)
InIncome		−0.281 **	−0.244 ***	−0.192 ***	−0.160 ***	0.061 *	−0.074 ***
		(0.135)	(0.087)	(0.059)	(0.041)	(0.037)	(0.028)
InDensity		−0.094 **	0.060 **	0.094 ***	0.071 ***	0.033 ***	0.058 ***
		(0.044)	(0.024)	(0.014)	(0.009)	(0.009)	(0.007)
Incons		2.098	4.357 ***	4.503 ***	3.932 ***	1.427 ***	3.225 ***
		(1.557)	(0.907)	(0.572)	(0.375)	(0.543)	(0.252)
R^2^		0.862	0.851	0.853	0.839	0.764	0.812
Observation		195	390	780	1651	1651	3302

*** *p* < 0.01, **p < 0.05, * *p* < 0.1.

**Table 7:** Robustness tests of income, density, and population coefficients with four additional subgroups.

	Subgroups	Top 15	Top 20	Top 30	Top 50	Top 60	Top 100	Top 127	Top 200	Bottom 127	All 254
Variables											
Income		−0.783 ***	−0.766 ***	−0.553 ***	−0.545 ***	−0.501 ***	−0.244 ***	−0.208 ***	−0.180 ***	0.157 **	−0.074 ***
Density		0.119	0.058	0.061 *	0.030	0.049 **	0.035 **	0.053 ***	0.030 ***	0.142 ***	0.058 ***
Population		0.261 ***	0.143 ***	0.027	−0.008	−0.034	−0.005	−0.009	−0.023 **	0.024	−0.018 **
Observation		195	260	390	650	780	1300	1651	2600	1651	3302

*** *p* < 0.01, ** *p* < 0.05, * *p* < 0.1.

**Table 8:** Threshold regression of PM_2.5_ concentrations for 254 cities by population size.

	In_PM_2.5_	Coef.	Std. Err.	z	*P* > z	95% Conf.	Interval]
Region1 (94 cities)	InPopulation	0.045 **	0.016	2.82	0.005	0.014	0.076
	InIncome	0.052 *	0.025	2.08	0.037	0.003	0.101
	InDensity	0.037 ***	0.005	6.89	0.000	0.027	0.048
	Incons	1.228 ***	0.305	4.03	0.000	0.630	1.823
Region2 (160 cities)	InPopulation	0.012	0.010	1.22	0.223	−0.007	0.031
	InIncome	−0.204 ***	0.027	−7.56	0.000	−0.257	−0.151
	InDensity	0.069 ***	0.006	11.33	0.000	0.057	0.081
	Incons	4.098 ***	0.253	16.18	0.000	3.601	4.594

Note: number of threshold = 1, threshold variable: InPopulation, threshold value of population = In13.551506 or 767,970 inhabitants, SSR = 145.5654, BIC = −10,250. *** *p* < 0.01, ** *p* < 0.05, * *p* < 0.1.

**Table 9:** Summary table of income, density, and population coefficients (2001–2013).

	Influencing Factors	Income		Density		Population	
		Average	Coefficient	Average	Coefficient	Average	Coefficient
Subgroups		(in $)		(in Persons per km^2^)		(in Million Inhabitants)	
	Bottom 127	30,007	0.157 **	202	0.142 ***	0.381	0.024
	All 254	37,772	−0.074 ***	704	0.058 ***	1.345	−0.018 **
	Top 127	45,537	−0.208 ***	1206	0.053 ***	2.318	−0.009
	Top 60	53,156	−0.501 ***	1919	0.049 **	3.990	−0.034
	Top 30	58,739	−0.553 ***	2835	0.061 *	6.415	0.027
	Top 15	63,152	−0.783 ***	4010	0.119	9.775	0.261 ***

*** *p* < 0.01, **p < 0.05, * *p* < 0.1.

## Data Availability

Data sources are reported in our Method and Data section. Data are available from these sources.

## Appendix A

**Table A1:** Ranking of 254 cities into 5 subgroups by income per capita.

Rank	Cities	gdp/cap	Population	Density	PM_2.5_
1	USA: San Francisco (Greater)	84,921	6,457,022	1285	11
2	USA: Boston	73,186	4,276,297	2343	8
3	USA: Houston	72,001	6,422,530	285	9
4	USA: New York (Greater)	70,399	20,000,933	1757	10
5	USA: New Haven	69,899	1,807,423	1068	10
6	DE: Ingolstadt	68,518	463,060	171	18
7	USA: Washington (Greater)	68,073	8,794,922	561	10
8	USA: Hartford	64,337	1,216,966	585	9
9	USA: Philadelphia (Greater)	61,836	6,407,666	756	10
10	DE: Dusseldorf	61,698	1,511,967	1708	14
11	USA: Minneapolis	61,415	3,405,918	238	10
12	USA: Portland	61,332	2,209,459	201	6
13	FR: Paris	61,301	11,866,785	1186	17
14	USA: Dallas	60,787	6,980,428	272	10
**Top 15**	**USA: Denver**	**60,752**	**2,696,308**	**226**	**8**
16	USA: Indianapolis	60,360	1,938,160	232	11
17	DE: Frankfurt am Main	60,351	2,544,366	648	15
18	USA: Tulsa	59,447	1,002,698	59	10
19	USA: Milwaukee	58,426	1,571,740	485	10
20	USA: Chicago	58,099	9,548,402	1089	11
21	USA: Nashville	58,031	294,618	427	10
22	USA: New Orleans	56,524	1,219,579	150	8
23	DE: Stuttgart	56,386	2,648,143	802	16
24	UK: London	55,954	11,544,026	2897	15
25	DE: Heilbronn	55,901	441,943	402	17
26	USA: East Baton Rouge	55,885	819,304	84	10
27	USA: Austin	55,841	1,894,164	257	8
28	USA: Charlotte	55,486	1,839,138	343	9
29	USA: Columbus	55,401	1,935,123	192	12
**Top 30**	**UK: Aberdeen**	**54,812**	**484,840**	**77**	**6**
31	USA: Cincinnati	54,682	2,084,836	268	11
32	USA: Atlanta	52,745	5,183,715	465	10
33	DE: Hamburg	52,487	3,143,783	487	11
34	USA: Jackson (MO)	52,441	1,977,173	124	9
35	USA: Richmond (Greater)	51,870	1,112,531	101	9
36	DE: Bonn	51,515	889,551	797	13
37	USA: Salt Lake	51,295	1,539,116	54	9
38	DE: Karlsruhe	51,240	722,801	667	16
39	DE: Wiesbaden	51,140	453,599	559	14
40	USA: Pittsburgh	50,378	1,441,884	646	11
41	USA: Oklahoma	50,025	1,281,128	120	9
42	DE: Regensburg	50,009	436,621	178	18
43	DE: Mainz	49,981	405,874	671	15
44	USA: Lancaster (NE)	49,898	328,854	110	11
45	USA: St. Louis	49,457	2,596,184	237	10
46	DE: Mannheim-Ludwigshafen	49,358	1,145,686	661	16
47	DE: Muenster	49,276	512,138	461	12
48	UK: Guildford	49,131	263,440	764	12
49	USA: Detroit (Greater)	48,811	4,360,382	885	11
50	DE: Koblenz	48,496	319,944	392	13
51	DE: Braunschweig-Salzgitter Wolfsburg	48,491	977,157	279	13
52	USA: Memphis	47,895	1,302,172	146	10
53	DE: Ulm	47,753	470,839	263	16
54	DE: Darmstadt	46,621	434,462	661	16
55	ES: Vitoria	46,600	264,719	200	10
56	USA: Virginia Beach	46,383	1,165,789	286	9
57	USA: Rochester (NY)	46,221	857,051	320	10
58	USA: Albany	45,650	976,721	118	8
59	FR: Lyon	45,114	1,958,191	622	17
**Top 60**	**USA: Sacramento**	**44,680**	**2,213,564**	**478**	**11**
61	DE: Hannover	44,588	1,267,062	458	12
62	DE: Schweinfurt	44,541	267,890	136	16
63	JPN: Toyohashi	44,353	665,226	961	14
64	UK: Oxford	44,033	527,670	281	13
65	USA: Erie (NY)	43,694	1,136,993	823	11
66	DE: Aschaffenburg	43,603	368,348	261	16
67	USA: Charleston	43,545	711,407	152	9
68	USA: Tuscaloosa	43,490	244,054	43	11
69	USA: Montgomery (OH)	43,485	697,435	646	11
70	USA: Phoenix	43,099	4,390,565	315	12
71	USA: Lafayette	43,007	427,049	54	12
72	DE: Reutlingen	42,986	273,578	272	16
73	UK: Edinburgh	42,842	849,720	582	9
74	USA: Miami (Greater)	42,808	6,014,211	3413	7
75	JPN: Tokyo	42,785	35,221,137	8635	16
76	USA: Providence	42,772	969,960	980	9
77	USA: Roanoke	42,757	311,993	65	8
78	ES: Barcelona	42,739	4,019,011	2076	14
79	FR: Toulouse	42,275	1,277,646	267	12
80	DE: Offenburg	42,147	412,179	232	15
81	ES: Madrid	42,105	6,379,915	991	10
82	DE: Bremen	42,075	1,230,691	221	12
83	JPN: Toyota	41,974	8,498,701	2403	13
84	USA: Allen	41,952	396,450	453	10
85	DE: Heidelberg	41,759	677,291	639	16
86	USA: San Antonio	41,750	2,298,261	122	8
87	USA: Las Vegas	41,274	2,074,253	28	7
88	DE: Wurzburg	41,216	497,551	168	16
89	UK: Bristol	40,865	913,519	1048	14
90	DE: Kassel	40,851	427,403	331	14
91	DE: Saarbrucken	40,635	800,458	586	14
92	USA: Hamilton (TN)	40,424	542,036	134	11
93	UK: Cambridge	40,261	360,154	232	13
94	DE: Augsburg	40,217	639,038	345	17
95	JPN: Hamamatsu	40,014	957,085	681	13
96	DE: Freiburg im Breisgau	39,783	623,036	303	15
97	JPN: Kanazawa	39,658	684,018	722	13
98	USA: Jacksonville	39,619	1,485,547	132	9
99	JPN: Fujieda	39,605	457,650	1069	14
100	JPN: Numazu	39,600	553,358	1100	13
101	DE: Iserlohn	39,246	420,986	451	12
102	JPN: Yokkaichi	38,989	1,058,231	1145	14
103	DE: Pforzheim	38,695	307,352	536	15
104	USA: Albuquerque	38,603	929,424	28	7
105	USA: Knox	38,534	463,248	723	9
106	DE: Aachen	38,469	539,521	995	13
107	ES: Pamplona	38,280	362,229	241	11
108	JPN: Utsunomiya	38,171	882,046	646	14
109	DE: Siegen	38,160	405,088	244	12
110	DE: Osnabruck	38,113	506,726	239	12
111	DE: Paderborn	37,865	298,853	281	12
112	DE: Rosenheim	37,486	307,074	213	18
113	USA: Spokane	37,376	501,584	51	8
114	UK: Southampton	37,211	664,608	481	13
115	JPN: Toyama	37,118	593,754	828	12
116	FR: Nice	36,979	824,441	353	16
117	DE: Kiel	36,979	632,735	195	10
118	ES: Bilbao	36,936	1,033,172	770	11
119	JPN: Fukui	36,794	547,512	306	14
120	JPN: Morioka	36,463	413,105	178	10
121	FR: Nantes	36,454	915,985	348	13
122	FR: Dijon	36,390	402,912	111	15
123	JPN: Mito	36,331	703,770	681	14
124	DE: Berlin	36,248	4,950,913	302	14
125	DE: Oldenburg (Oldenburg)	36,096	402,152	224	11
126	JPN: Sendai	36,011	1,464,672	1435	13
**Top 127**	**FR: Rennes**	**35,966**	**701,153**	**219**	**13**
128	FR: Reims	35,964	320,879	137	15
129	USA: Tallahassee	35,883	373,212	81	9
130	FR: Strasbourg	35,874	771,559	449	16
131	JPN: Hitachi	35,740	316,365	420	13
132	UK: Northampton	35,711	457,540	313	13
133	DE: Gottingen	35,692	383,137	169	14
134	JPN: Tokushima	35,601	569,456	620	15
135	DE: Wetzlar	35,552	251,578	255	13
136	JPN: Hiroshima	35,436	1,432,615	3149	17
137	FR: Bordeaux	35,367	1,174,012	235	12
138	USA: Fresno (Greater)	35,140	1,105,606	198	15
139	JPN: Kurashiki	34,908	1,516,388	962	16
140	JPN: Takamatsu	34,900	562,614	1285	15
141	FR: Pau	34,751	267,702	146	11
142	FR: Orleans	34,633	424,619	164	14
143	FR: Clermont-Ferrand	34,545	476,713	202	13
144	JPN: Niigata	34,529	805,385	1446	14
145	ES: Palma de Mallorca	34,502	643,352	355	11
146	JPN: Wakayama	34,473	541,730	1180	15
147	DE: Lubeck	34,352	407,813	295	11
148	FR: Rouen	34,337	689,626	313	16
149	JPN: Kofu	34,222	586,614	433	12
150	JPN: Kurume	34,202	409,982	1898	19
151	FR: Grenoble	34,031	661,221	282	16
152	JPN: Nagano	33,981	572,858	732	12
153	DE: Flensburg	33,897	274,656	138	9
154	UK: Derby	33,806	472,015	783	14
155	JPN: Koriyama	33,754	518,284	513	12
156	UK: Manchester	33,644	3,246,448	1762	13
157	DE: Leipzig	33,626	978,997	266	15
158	JPN: Matsumoto	33,581	426,101	477	11
159	JPN: Fukushima	33,569	449,041	548	12
160	DE: Erfurt	33,561	519,509	201	15
161	USA: Montgomery (AL)	33,516	451,815	39	11
162	FR: Le Havre	33,328	297,916	537	16
163	USA: Lubbock	33,053	351,009	29	6
164	DE: Trier	33,037	248,567	227	13
165	DE: Halle an der Saale	33,026	420,210	292	14
166	DE: Magdeburg	33,017	496,349	125	14
167	UK: Brighton and Hove	32,843	440,222	1502	16
168	JPN: Kitakyushu	32,749	1,332,183	2680	20
169	UK: Glasgow	32,740	1,790,510	780	10
170	JPN: Oita	32,635	732,952	568	17
171	JPN: Fukuoka	32,630	2,680,715	5918	21
172	DE: Rostock	32,549	412,399	121	11
173	JPN: Matsuyama	32,249	625,918	6137	17
174	FR: Caen	32,169	434,109	211	14
175	FR: Valenciennes	32,133	358,729	687	16
176	FR: Avignon	32,098	318,245	505	13
177	JPN: Asahikawa	32,090	388,628	232	9
178	FR: Annecy	32,023	272,588	256	15
179	UK: Ipswich	31,738	349,520	235	13
180	FR: Tours	31,733	460,093	183	14
181	FR: Lille	31,700	1,366,909	1647	16
182	FR: Poitiers	31,591	266,275	118	13
183	FR: Montpellier	31,425	668,380	372	12
184	JPN: Obihiro	31,404	262,830	150	9
185	DE: Dresden	31,378	1,327,534	241	16
186	JPN: Himeji	31,342	720,892	1139	16
187	FR: Le Mans	31,328	355,467	171	15
188	JPN: Sapporo	31,201	2,192,770	1629	11
189	UK: Leicester	31,141	849,964	728	13
190	ES: Valladolid	30,983	414,196	431	8
191	UK: Leeds	30,926	2,550,810	740	12
192	JPN: Yamagata	30,785	422,839	571	13
193	UK: Norwich	30,726	388,299	266	12
194	DE: Kaiserslautern	30,696	273,554	226	14
195	DE: Bremerhaven	30,604	307,055	149	11
196	JPN: Aomori	30,530	309,601	585	11
197	FR: Nancy	30,446	474,407	176	14
198	FR: Dunkerque	30,390	273,513	425	17
199	JPN: Hakodate	30,317	345,811	562	10
200	JPN: Hachinohe	30,196	324,182	405	10
201	USA: El Paso (TX)	30,194	833,522	70	8
202	JPN: Akita	30,093	399,793	624	13
203	JPN: Miyazaki	29,868	493,598	639	15
204	JPN: Kumamoto	29,811	1,130,440	753	20
205	FR: Amiens	29,772	309,154	145	17
206	DE: Zwickau	29,645	329,603	390	15
207	UK: Nottingham	29,576	884,410	1068	13
208	UK: Dundee City	29,547	264,390	121	8
209	FR: Mulhouse	29,505	407,282	414	16
210	FR: Angers	29,380	406,872	227	14
211	DE: Hildesheim	29,355	276,440	248	12
212	FR: Besancon	29,345	270,164	138	15
213	JPN: Nagasaki	29,342	641,205	1869	17
214	UK: Liverpool	29,312	1,178,689	6204	13
215	DE: Schwerin	29,287	303,031	64	11
216	JPN: Kagoshima	29,173	715,775	1473	15
217	FR: Limoges	29,160	307,992	114	12
218	FR: Brest	29,048	365,055	336	10
219	FR: Saint-Etienne	28,911	526,369	425	13
220	UK: Plymouth	28,849	396,686	193	11
221	UK: Exeter	28,828	460,870	192	11
222	UK: Blackburn with Darwen	28,709	285,594	489	10
223	UK: Newcastle upon Tyne	28,450	1,152,859	230	9
224	ES: Oviedo	28,336	304,133	407	8
225	JPN: Kochi	28,335	513,465	342	16
226	ES: Valencia	28,298	1,663,496	1243	12
227	ES: Santander	27,833	372,909	608	9
228	DE: Neubrandenburg	27,794	278,044	48	12
229	UK: Cardiff	27,691	767,542	1095	13
230	ES: Santa Cruz de Tenerife	27,235	492,820	1311	12
231	DE: Gorlitz	27,186	264,402	130	17
232	FR: Metz	27,067	368,383	247	14
233	UK: Kingston upon Hull	26,879	593,260	246	11
234	UK: Lincoln	26,721	296,097	143	11
235	UK: Stoke-on-Trent	26,405	472,866	822	11
236	UK: Sheffield	26,220	1,154,133	4197	12
237	UK: Blackpool	26,076	326,318	1966	13
238	ES: Murcia	26,062	591,669	1992	11
239	UK: Middlesbrough	25,983	467,304	1907	11
240	UK: Swansea	25,891	379,975	858	12
241	JPN: Naha	25,838	1,170,320	3715	8
242	FR: Toulon	25,681	553,594	818	13
243	UK: Colchester	25,622	317,030	932	14
244	ES: Gijon	25,587	296,163	863	9
245	ES: Las Palmas	25,354	620,841	1127	13
246	ES: Vigo	25,251	533,676	430	9
247	ES: Elche/Elx	25,221	249,200	4224	12
248	FR: Perpignan	25,092	389,016	326	11
249	FR: Nimes	24,987	338,177	414	11
250	ES: Seville	24,789	1,498,774	372	13
251	ES: Alicante	23,321	439,642	3891	12
252	ES: Marbella	23,226	285,326	509	13
253	ES: Granada	22,719	538,657	402	11
**Bottom 127**	**ES: Cordoba**	**22,057**	**355,038**	**577**	**11**

**Table A2:** Ranking of 254 cities into 5 subgroups by population density.

Rank	Cities	Density	gdp/Capita	Population	PM_2.5_
1	JPN01: Tokyo	8635	42,785	35,221,137	16
2	UK006: Liverpool	6204	29,312	1,178,689	13
3	JPN25: Matsuyama	6137	32,249	625,918	17
4	JPN04: Fukuoka	5918	32,630	2,680,715	21
5	ES505: Elche/Elx	4224	25,221	249,200	12
6	UK010: Sheffield	4197	26,220	1,154,133	12
7	ES021: Alicante	3891	23,321	439,642	12
8	JPN10: Naha	3715	25,838	1,170,320	8
9	USA09: Miami (Greater)	3413	42,808	6,014,211	7
10	JPN08: Hiroshima	3149	35,436	1,432,615	17
11	UK001: London	2897	55,954	11,544,026	15
12	JPN09: Kitakyushu	2680	32,749	1,332,183	20
13	JPN03: Toyota	2403	41,974	8,498,701	13
14	USA11: Boston	2343	73,186	4,276,297	8
**Top 15**	**ES002: Barcelona**	**2076**	**42,739**	**4,019,011**	**14**
16	ES007: Murcia	1992	26,062	591,669	11
17	UK553: Blackpool	1966	26,076	326,318	13
18	UK559: Middlesbrough	1907	25,983	467,304	11
19	JPN42: Kurume	1898	34,202	409,982	19
20	JPN24: Nagasaki	1869	29,342	641,205	17
21	UK008: Manchester	1762	33,644	3,246,448	13
22	USA01: New York (Greater)	1757	70,399	20,000,933	10
23	DE011: Dusseldorf	1708	61,698	1,511,967	14
24	FR009: Lille	1647	31,700	1,366,909	16
25	JPN05: Sapporo	1629	31,201	2,192,770	11
26	UK515: Brighton and Hove	1502	32,843	440,222	16
27	JPN19: Kagoshima	1473	29,173	715,775	15
28	JPN15: Niigata	1446	34,529	805,385	14
29	JPN06: Sendai	1435	36,011	1,464,672	13
**Top 30**	**ES025: Santa Cruz de Tenerife**	**1311**	**27,235**	**492,820**	**12**
31	USA05: San Francisco (Greater)	1285	84,921	6,457,022	11
32	JPN28: Takamatsu	1285	34,900	562,614	15
33	ES003: Valencia	1243	28,298	1,663,496	12
34	FR001: Paris	1186	61,301	11,866,785	17
35	JPN33: Wakayama	1180	34,473	541,730	15
36	JPN12: Yokkaichi	1145	38,989	1,058,231	14
37	JPN20: Himeji	1139	31,342	720,892	16
38	ES008: Las Palmas	1127	25,354	620,841	13
39	JPN31: Numazu	1100	39,600	553,358	13
40	UK009: Cardiff	1095	27,691	767,542	13
41	USA03: Chicago	1089	58,099	9,548,402	11
42	JPN38: Fujieda	1069	39,605	457,650	14
43	USA27: New Haven	1068	69,899	1,807,423	10
44	UK029: Nottingham	1068	29,576	884,410	13
45	UK011: Bristol	1048	40,865	913,519	14
46	DE507: Aachen	995	38,469	539,521	13
47	ES001: Madrid	991	42,105	6,379,915	10
48	USA53: Providence	980	42,772	969,960	9
49	JPN07: Kurashiki	962	34,908	1,516,388	16
50	JPN23: Toyohashi	961	44,353	665,226	14
51	UK546: Colchester	932	25,622	317,030	14
52	USA13: Detroit (Greater)	885	48,811	4,360,382	11
53	ES023: Gijon	863	25,587	296,163	9
54	UK517: Swansea	858	25,891	379,975	12
55	JPN26: Toyama	828	37,118	593,754	12
56	USA44: Erie (NY)	823	43,694	1,136,993	11
57	UK027: Stoke-on-Trent	822	26,405	472,866	11
58	FR032: Toulon	818	25,681	553,594	13
59	DE007: Stuttgart	802	56,386	2,648,143	16
**Top 60**	**DE034: Bonn**	**797**	**51,515**	**889,551**	**13**
61	UK518: Derby	783	33,806	472,015	14
62	UK004: Glasgow	780	32,740	1,790,510	10
63	ES019: Bilbao	770	36,936	1,033,172	11
64	UK033: Guildford	764	49,131	263,440	12
65	USA06: Philadelphia (Greater)	756	61,836	6,407,666	10
66	JPN11: Kumamoto	753	29,811	1,130,440	20
67	UK003: Leeds	740	30,926	2,550,810	12
68	JPN29: Nagano	732	33,981	572,858	12
69	UK014: Leicester	728	31,141	849,964	13
70	USA97: Knox	723	38,534	463,248	9
71	JPN17: Kanazawa	722	39,658	684,018	13
72	FR034: Valenciennes	687	32,133	358,729	16
73	JPN14: Hamamatsu	681	40,014	957,085	13
74	JPN21: Mito	681	36,331	703,770	14
75	DE037: Mainz	671	49,981	405,874	15
76	DE035: Karlsruhe	667	51,240	722,801	16
77	DE084: Mannheim-Ludwigshafen	661	49,358	1,145,686	16
78	DE025: Darmstadt	661	46,621	434,462	16
79	DE005: Frankfurt am Main	648	60,351	2,544,366	15
80	USA41: Pittsburgh	646	50,378	1,441,884	11
81	JPN16: Utsunomiya	646	38,171	882,046	14
82	USA66: Montgomery (OH)	646	43,485	697,435	11
83	DE522: Heidelberg	639	41,759	677,291	16
84	JPN36: Miyazaki	639	29,868	493,598	15
85	JPN43: Akita	624	30,093	399,793	13
86	FR003: Lyon	622	45,114	1,958,191	17
87	JPN30: Tokushima	620	35,601	569,456	15
88	ES015: Santander	608	27,833	372,909	9
89	DE040: Saarbrucken	586	40,635	800,458	14
90	USA40: Hartford	585	64,337	1,216,966	9
91	JPN51: Aomori	585	30,530	309,601	11
92	UK007: Edinburgh	582	42,842	849,720	9
93	ES020: Cordoba	577	22,057	355,038	11
94	JPN41: Yamagata	571	30,785	422,839	13
95	JPN18: Oita	568	32,635	732,952	17
96	JPN48: Hakodate	562	30,317	345,811	10
97	USA04: Washington (Greater)	561	68,073	8,794,922	10
98	DE020: Wiesbaden	559	51,140	453,599	14
99	JPN39: Fukushima	548	33,569	449,041	12
100	FR012: Le Havre	537	33,328	297,916	16
101	DE533: Pforzheim	536	38,695	307,352	15
102	JPN34: Koriyama	513	33,754	518,284	12
103	ES533: Marbella	509	23,226	285,326	13
104	FR039: Avignon	505	32,098	318,245	13
105	UK557: Blackburn with Darwen	489	28,709	285,594	10
106	DE002: Hamburg	487	52,487	3,143,783	11
107	USA32: Milwaukee	485	58,426	1,571,740	10
108	UK520: Southampton	481	37,211	664,608	13
109	USA29: Sacramento	478	44,680	2,213,564	11
110	JPN40: Matsumoto	477	33,581	426,101	11
111	USA10: Atlanta	465	52,745	5,183,715	10
112	DE504: Muenster	461	49,276	512,138	12
113	DE013: Hannover	458	44,588	1,267,062	12
114	USA116: Allen	453	41,952	396,450	10
115	DE045: Iserlohn	451	39,246	420,986	12
116	FR006: Strasbourg	449	35,874	771,559	16
117	JPN27: Kofu	433	34,222	586,614	12
118	ES009: Valladolid	431	30,983	414,196	8
119	ES022: Vigo	430	25,251	533,676	9
120	USA145: Nashville	427	58,031	294,618	10
121	FR011: Saint-Etienne	425	28,911	526,369	13
122	FR042: Dunkerque	425	30,390	273,513	17
123	JPN50: Hitachi	420	35,740	316,365	13
124	FR040: Mulhouse	414	29,505	407,282	16
125	FR044: Nimes	414	24,987	338,177	11
126	ES013: Oviedo	407	28,336	304,133	8
**Top 127**	**JPN49: Hachinohe**	**405**	**30,196**	**324,182**	**10**
128	ES501: Granada	402	22,719	538,657	11
129	DE529: Heilbronn	402	55,901	441,943	17
130	DE042: Koblenz	392	48,496	319,944	13
131	DE544: Zwickau	390	29,645	329,603	15
132	ES004: Seville	372	24,789	1,498,774	13
133	FR010: Montpellier	372	31,425	668,380	12
134	ES010: Palma de Mallorca	355	34,502	643,352	11
135	FR205: Nice	353	36,979	824,441	16
136	FR008: Nantes	348	36,454	915,985	13
137	DE033: Augsburg	345	40,217	639,038	17
138	USA28: Charlotte	343	55,486	1,839,138	9
139	JPN35: Kochi	342	28,335	513,465	16
140	FR037: Brest	336	29,048	365,055	10
141	DE513: Kassel	331	40,851	427,403	14
142	FR043: Perpignan	326	25,092	389,016	11
143	USA56: Rochester (NY)	320	46,221	857,051	10
144	USA12: Phoenix	315	43,099	4,390,565	12
145	FR215: Rouen	313	34,337	689,626	16
146	UK528: Northampton	313	35,711	457,540	13
147	JPN32: Fukui	306	36,794	547,512	14
148	DE027: Freiburg im Breisgau	303	39,783	623,036	15
149	DE001: Berlin	302	36,248	4,950,913	14
150	DE510: Lubeck	295	34,352	407,813	11
151	DE018: Halle an der Saale	292	33,026	420,210	14
152	USA43: Virginia Beach	286	46,383	1,165,789	9
153	USA08: Houston	285	72,001	6,422,530	9
154	FR026: Grenoble	282	34,031	661,221	16
155	UK560: Oxford	281	44,033	527,670	13
156	DE523: Paderborn	281	37,865	298,853	12
157	DE083: Braunschweig-Salzgitter Wolfsburg	279	48,491	977,157	13
158	USA07: Dallas	272	60,787	6,980,428	10
159	DE537: Reutlingen	272	42,986	273,578	16
160	USA21: Cincinnati	268	54,682	2,084,836	11
161	FR004: Toulouse	267	42,275	1,277,646	12
162	DE008: Leipzig	266	33,626	978,997	15
163	UK566: Norwich	266	30,726	388,299	12
164	DE532: Ulm	263	47,753	470,839	16
165	DE061: Aschaffenburg	261	43,603	368,348	16
166	USA30: Austin	257	55,841	1,894,164	8
167	FR048: Annecy	256	32,023	272,588	15
168	DE079: Wetzlar	255	35,552	251,578	13
169	DE542: Hildesheim	248	29,355	276,440	12
170	FR017: Metz	247	27,067	368,383	14
171	UK026: Kingston upon Hull	246	26,879	593,260	11
172	DE540: Siegen	244	38,160	405,088	12
173	DE009: Dresden	241	31,378	1,327,534	16
174	ES014: Pamplona	241	38,280	362,229	11
175	DE517: Osnabruck	239	38,113	506,726	12
176	USA15: Minneapolis	238	61,415	3,405,918	10
177	USA17: St. Louis	237	49,457	2,596,184	10
178	FR007: Bordeaux	235	35,367	1,174,012	12
179	UK569: Ipswich	235	31,738	349,520	13
180	USA25: Indianapolis	232	60,360	1,938,160	11
181	DE073: Offenburg	232	42,147	412,179	15
182	JPN44: Asahikawa	232	32,090	388,628	9
183	UK017: Cambridge	232	40,261	360,154	13
184	UK013: Newcastle upon Tyne	230	28,450	1,152,859	9
185	FR036: Angers	227	29,380	406,872	14
186	DE026: Trier	227	33,037	248,567	13
187	USA18: Denver	226	60,752	2,696,308	8
188	DE044: Kaiserslautern	226	30,696	273,554	14
189	DE520: Oldenburg (Oldenburg)	224	36,096	402,152	11
190	DE012: Bremen	221	42,075	1,230,691	12
191	FR013: Rennes	219	35,966	701,153	13
192	DE069: Rosenheim	213	37,486	307,074	18
193	FR023: Caen	211	32,169	434,109	14
194	FR022: Clermont-Ferrand	202	34,545	476,713	13
195	USA20: Portland	201	61,332	2,209,459	6
196	DE032: Erfurt	201	33,561	519,509	15
197	ES012: Vitoria	200	46,600	264,719	10
198	USA45: Fresno (Greater)	198	35,140	1,105,606	15
199	DE039: Kiel	195	36,979	632,735	10
200	UK516: Plymouth	193	28,849	396,686	11
201	USA31: Columbus	192	55,401	1,935,123	12
202	UK018: Exeter	192	28,828	460,870	11
203	FR035: Tours	183	31,733	460,093	14
204	DE028: Regensburg	178	50,009	436,621	18
205	JPN37: Morioka	178	36,463	413,105	10
206	FR016: Nancy	176	30,446	474,407	14
207	DE534: Ingolstadt	171	68,518	463,060	18
208	FR038: Le Mans	171	31,328	355,467	15
209	DE021: Gottingen	169	35,692	383,137	14
210	DE524: Wurzburg	168	41,216	497,551	16
211	FR019: Orleans	164	34,633	424,619	14
212	USA69: Charleston	152	43,545	711,407	9
213	USA42: New Orleans	150	56,524	1,219,579	8
214	JPN53: Obihiro	150	31,404	262,830	9
215	DE527: Bremerhaven	149	30,604	307,055	11
216	USA37: Memphis	146	47,895	1,302,172	10
217	FR045: Pau	146	34,751	267,702	11
218	FR014: Amiens	145	29,772	309,154	17
219	UK019: Lincoln	143	26,721	296,097	11
220	DE052: Flensburg	138	33,897	274,656	9
221	FR025: Besancon	138	29,345	270,164	15
222	FR018: Reims	137	35,964	320,879	15
223	DE077: Schweinfurt	136	44,541	267,890	16
224	USA83: Hamilton (TN)	134	40,424	542,036	11
225	USA33: Jacksonville	132	39,619	1,485,547	9
226	DE074: Gorlitz	130	27,186	264,402	17
227	DE019: Magdeburg	125	33,017	496,349	14
228	USA24: Jackson (MO)	124	52,441	1,977,173	9
229	USA19: San Antonio	122	41,750	2,298,261	8
230	DE043: Rostock	121	32,549	412,399	11
231	UK550: Dundee City	121	29,547	264,390	8
232	USA39: Oklahoma	120	50,025	1,281,128	9
233	USA52: Albany	118	45,650	976,721	8
234	FR021: Poitiers	118	31,591	266,275	13
235	FR024: Limoges	114	29,160	307,992	12
236	FR020: Dijon	111	36,390	402,912	15
237	USA132: Lancaster (NE)	110	49,898	328,854	11
238	USA46: Richmond (Greater)	101	51,870	1,112,531	9
239	USA60: East Baton Rouge	84	55,885	819,304	10
240	USA119: Tallahassee	81	35,883	373,212	9
241	UK016: Aberdeen	77	54,812	484,840	6
242	USA59: El Paso (TX)	70	30,194	833,522	8
243	USA135: Roanoke	65	42,757	311,993	8
244	DE031: Schwerin	64	29,287	303,031	11
245	USA51: Tulsa	59	59,447	1,002,698	10
246	USA34: Salt Lake	54	51,295	1,539,116	9
247	USA108: Lafayette	54	43,007	427,049	12
248	USA89: Spokane	51	37,376	501,584	8
249	DE064: Neubrandenburg	48	27,794	278,044	12
250	USA162: Tuscaloosa	43	43,490	244,054	11
251	USA96: Montgomery (AL)	39	33,516	451,815	11
252	USA126: Lubbock	29	33,053	351,009	6
253	USA22: Las Vegas	28	41,274	2,074,253	7
**Bottom 127**	**USA54: Albuquerque**	**28**	**38,603**	**929,424**	**7**

**Table A3:** Ranking of 254 cities into 5 subgroups by population.

Rank	Cities	Population	Density	gdp/Capita	PM_2.5_
1	JPN: Tokyo	35,221,137	8635	42,785	16
2	USA: New York (Greater)	20,000,933	1757	70,399	10
3	FR: Paris	11,866,785	1186	61,301	17
4	UK: London	11,544,026	2897	55,954	15
5	USA: Chicago	9,548,402	1089	58,099	11
6	USA: Washington (Greater)	8,794,922	561	68,073	10
7	JPN: Toyota	8,498,701	2403	41,974	13
8	USA: Dallas	6,980,428	272	60,787	10
9	USA: San Francisco (Greater)	6,457,022	1285	84,921	11
10	USA: Houston	6,422,530	285	72,001	9
11	USA: Philadelphia (Greater)	6,407,666	756	61,836	10
12	ES: Madrid	6,379,915	991	42,105	10
13	USA: Miami (Greater)	6,014,211	3413	42,808	7
14	USA: Atlanta	5,183,715	465	52,745	10
**Top 15**	**DE: Berlin**	**4,950,913**	**302**	**36,248**	**14**
16	USA: Phoenix	4,390,565	315	43,099	12
17	USA: Detroit (Greater)	4,360,382	885	48,811	11
18	USA: Boston	4,276,297	2343	73,186	8
19	ES: Barcelona	4,019,011	2076	42,739	14
20	USA: Minneapolis	3,405,918	238	61,415	10
21	UK: Manchester	3,246,448	1762	33,644	13
22	DE: Hamburg	3,143,783	487	52,487	11
23	USA: Denver	2,696,308	226	60,752	8
24	JPN: Fukuoka	2,680,715	5918	32,630	21
25	DE: Stuttgart	2,648,143	802	56,386	16
26	USA: St. Louis	2,596,184	237	49,457	10
27	UK: Leeds	2,550,810	740	30,926	12
28	DE: Frankfurt am Main	2,544,366	648	60,351	15
29	USA: San Antonio	2,298,261	122	41,750	8
**Top 30**	**USA: Sacramento**	**2,213,564**	**478**	**44,680**	**11**
31	USA: Portland	2,209,459	201	61,332	6
32	JPN: Sapporo	2,192,770	1629	31,201	11
33	USA: Cincinnati	2,084,836	268	54,682	11
34	USA: Las Vegas	2,074,253	28	41,274	7
35	USA: Jackson (MO)	1,977,173	124	52,441	9
36	FR: Lyon	1,958,191	622	45,114	17
37	USA: Indianapolis	1,938,160	232	60,360	11
38	USA: Columbus	1,935,123	192	55,401	12
39	USA: Austin	1,894,164	257	55,841	8
40	USA: Charlotte	1,839,138	343	55,486	9
41	USA: New Haven	1,807,423	1068	69,899	10
42	UK: Glasgow	1,790,510	780	32,740	10
43	ES: Valencia	1,663,496	1243	28,298	12
44	USA: Milwaukee	1,571,740	485	58,426	10
45	USA: Salt Lake	1,539,116	54	51,295	9
46	JPN: Kurashiki	1,516,388	962	34,908	16
47	DE: Dusseldorf	1,511,967	1708	61,698	14
48	ES: Seville	1,498,774	372	24,789	13
49	USA: Jacksonville	1,485,547	132	39,619	9
50	JPN: Sendai	1,464,672	1435	36,011	13
51	USA: Pittsburgh	1,441,884	646	50,378	11
52	JPN: Hiroshima	1,432,615	3149	35,436	17
53	FR: Lille	1,366,909	1647	31,700	16
54	JPN: Kitakyushu	1,332,183	2680	32,749	20
55	DE: Dresden	1,327,534	241	31,378	16
56	USA: Memphis	1,302,172	146	47,895	10
57	USA: Oklahoma	1,281,128	120	50,025	9
58	FR: Toulouse	1,277,646	267	42,275	12
59	DE: Hannover	1,267,062	458	44,588	12
**Top 60**	**DE: Bremen**	**1,230,691**	**221**	**42,075**	**12**
61	USA: New Orleans	1,219,579	150	56,524	8
62	USA: Hartford	1,216,966	585	64,337	9
63	UK: Liverpool	1,178,689	6204	29,312	13
64	FR: Bordeaux	1,174,012	235	35,367	12
65	JPN: Naha	1,170,320	3715	25,838	8
66	USA: Virginia Beach	1,165,789	286	46,383	9
67	UK: Sheffield	1,154,133	4197	26,220	12
68	UK: Newcastle upon Tyne	1,152,859	230	28,450	9
69	DE: Mannheim-Ludwigshafen	1,145,686	661	49,358	16
70	USA: Erie (NY)	1,136,993	823	43,694	11
71	JPN: Kumamoto	1,130,440	753	29,811	20
72	USA: Richmond (Greater)	1,112,531	101	51,870	9
73	USA: Fresno (Greater)	1,105,606	198	35,140	15
74	JPN: Yokkaichi	1,058,231	1145	38,989	14
75	ES: Bilbao	1,033,172	770	36,936	11
76	USA: Tulsa	1,002,698	59	59,447	10
77	DE: Leipzig	978,997	266	33,626	15
78	DE: Braunschweig-Salzgitter Wolfsburg	977,157	279	48,491	13
79	USA: Albany	976,721	118	45,650	8
80	USA: Providence	969,960	980	42,772	9
81	JPN: Hamamatsu	957,085	681	40,014	13
82	USA: Albuquerque	929,424	28	38,603	7
83	FR: Nantes	915,985	348	36,454	13
84	UK: Bristol	913,519	1048	40,865	14
85	DE: Bonn	889,551	797	51,515	13
86	UK: Nottingham	884,410	1068	29,576	13
87	JPN: Utsunomiya	882,046	646	38,171	14
88	USA: Rochester (NY)	857,051	320	46,221	10
89	UK: Leicester	849,964	728	31,141	13
90	UK: Edinburgh	849,720	582	42,842	9
91	USA: El Paso (TX)	833,522	70	30,194	8
92	FR: Nice	824,441	353	36,979	16
93	USA: East Baton Rouge	819,304	84	55,885	10
94	JPN: Niigata	805,385	1446	34,529	14
95	DE: Saarbrucken	800,458	586	40,635	14
96	FR: Strasbourg	771,559	449	35,874	16
97	UK: Cardiff	767,542	1095	27,691	13
98	JPN: Oita	732,952	568	32,635	17
99	DE: Karlsruhe	722,801	667	51,240	16
100	JPN: Himeji	720,892	1139	31,342	16
101	JPN: Kagoshima	715,775	1473	29,173	15
102	USA: Charleston	711,407	152	43,545	9
103	JPN: Mito	703,770	681	36,331	14
104	FR: Rennes	701,153	219	35,966	13
105	USA: Montgomery (OH)	697,435	646	43,485	11
106	FR: Rouen	689,626	313	34,337	16
107	JPN: Kanazawa	684,018	722	39,658	13
108	DE: Heidelberg	677,291	639	41,759	16
109	FR: Montpellier	668,380	372	31,425	12
110	JPN: Toyohashi	665,226	961	44,353	14
111	UK: Southampton	664,608	481	37,211	13
112	FR: Grenoble	661,221	282	34,031	16
113	ES: Palma de Mallorca	643,352	355	34,502	11
114	JPN: Nagasaki	641,205	1869	29,342	17
115	DE: Augsburg	639,038	345	40,217	17
116	DE: Kiel	632,735	195	36,979	10
117	JPN: Matsuyama	625,918	6137	32,249	17
118	DE: Freiburg im Breisgau	623,036	303	39,783	15
119	ES: Las Palmas	620,841	1127	25,354	13
120	JPN: Toyama	593,754	828	37,118	12
121	UK: Kingston upon Hull	593,260	246	26,879	11
122	ES: Murcia	591,669	1992	26,062	11
123	JPN: Kofu	586,614	433	34,222	12
124	JPN: Nagano	572,858	732	33,981	12
125	JPN: Tokushima	569,456	620	35,601	15
126	JPN: Takamatsu	562,614	1285	34,900	15
**Top 127**	**FR: Toulon**	**553,594**	**818**	**25,681**	**13**
128	JPN: Numazu	553,358	1100	39,600	13
129	JPN: Fukui	547,512	306	36,794	14
130	USA: Hamilton (TN)	542,036	134	40,424	11
131	JPN: Wakayama	541,730	1180	34,473	15
132	DE: Aachen	539,521	995	38,469	13
133	ES: Granada	538,657	402	22,719	11
134	ES: Vigo	533,676	430	25,251	9
135	UK: Oxford	527,670	281	44,033	13
136	FR: Saint-Etienne	526,369	425	28,911	13
137	DE: Erfurt	519,509	201	33,561	15
138	JPN: Koriyama	518,284	513	33,754	12
139	JPN: Kochi	513,465	342	28,335	16
140	DE: Muenster	512,138	461	49,276	12
141	DE: Osnabruck	506,726	239	38,113	12
142	USA: Spokane	501,584	51	37,376	8
143	DE: Wurzburg	497,551	168	41,216	16
144	DE: Magdeburg	496,349	125	33,017	14
145	JPN: Miyazaki	493,598	639	29,868	15
146	ES: Santa Cruz de Tenerife	492,820	1311	27,235	12
147	UK: Aberdeen	484,840	77	54,812	6
148	FR: Clermont-Ferrand	476,713	202	34,545	13
149	FR: Nancy	474,407	176	30,446	14
150	UK: Stoke-on-Trent	472,866	822	26,405	11
151	UK: Derby	472,015	783	33,806	14
152	DE: Ulm	470,839	263	47,753	16
153	UK: Middlesbrough	467,304	1907	25,983	11
154	USA: Knox	463,248	723	38,534	9
155	DE: Ingolstadt	463,060	171	68,518	18
156	UK: Exeter	460,870	192	28,828	11
157	FR: Tours	460,093	183	31,733	14
158	JPN: Fujieda	457,650	1069	39,605	14
159	UK: Northampton	457,540	313	35,711	13
160	DE: Wiesbaden	453,599	559	51,140	14
161	USA: Montgomery (AL)	451,815	39	33,516	11
162	JPN: Fukushima	449,041	548	33,569	12
163	DE: Heilbronn	441,943	402	55,901	17
164	UK: Brighton and Hove	440,222	1502	32,843	16
165	ES: Alicante	439,642	3891	23,321	12
166	DE: Regensburg	436,621	178	50,009	18
167	DE: Darmstadt	434,462	661	46,621	16
168	FR: Caen	434,109	211	32,169	14
169	DE: Kassel	427,403	331	40,851	14
170	USA: Lafayette	427,049	54	43,007	12
171	JPN: Matsumoto	426,101	477	33,581	11
172	FR: Orleans	424,619	164	34,633	14
173	JPN: Yamagata	422,839	571	30,785	13
174	DE: Iserlohn	420,986	451	39,246	12
175	DE: Halle an der Saale	420,210	292	33,026	14
176	ES: Valladolid	414,196	431	30,983	8
177	JPN: Morioka	413,105	178	36,463	10
178	DE: Rostock	412,399	121	32,549	11
179	DE: Offenburg	412,179	232	42,147	15
180	JPN: Kurume	409,982	1898	34,202	19
181	DE: Lubeck	407,813	295	34,352	11
182	FR: Mulhouse	407,282	414	29,505	16
183	FR: Angers	406,872	227	29,380	14
184	DE: Mainz	405,874	671	49,981	15
185	DE: Siegen	405,088	244	38,160	12
186	FR: Dijon	402,912	111	36,390	15
187	DE: Oldenburg (Oldenburg)	402,152	224	36,096	11
188	JPN: Akita	399,793	624	30,093	13
189	UK: Plymouth	396,686	193	28,849	11
190	USA: Allen	396,450	453	41,952	10
191	FR: Perpignan	389,016	326	25,092	11
192	JPN: Asahikawa	388,628	232	32,090	9
193	UK: Norwich	388,299	266	30,726	12
194	DE: Gottingen	383,137	169	35,692	14
195	UK: Swansea	379,975	858	25,891	12
196	USA: Tallahassee	373,212	81	35,883	9
197	ES: Santander	372,909	608	27,833	9
198	FR: Metz	368,383	247	27,067	14
199	DE: Aschaffenburg	368,348	261	43,603	16
200	FR: Brest	365,055	336	29,048	10
201	ES: Pamplona	362,229	241	38,280	11
202	UK: Cambridge	360,154	232	40,261	13
203	FR: Valenciennes	358,729	687	32,133	16
204	FR: Le Mans	355,467	171	31,328	15
205	ES: Cordoba	355,038	577	22,057	11
206	USA: Lubbock	351,009	29	33,053	6
207	UK: Ipswich	349,520	235	31,738	13
208	JPN: Hakodate	345,811	562	30,317	10
209	FR: Nimes	338,177	414	24,987	11
210	DE: Zwickau	329,603	390	29,645	15
211	USA: Lancaster (NE)	328,854	110	49,898	11
212	UK: Blackpool	326,318	1966	26,076	13
213	JPN: Hachinohe	324,182	405	30,196	10
214	FR: Reims	320,879	137	35,964	15
215	DE: Koblenz	319,944	392	48,496	13
216	FR: Avignon	318,245	505	32,098	13
217	UK: Colchester	317,030	932	25,622	14
218	JPN: Hitachi	316,365	420	35,740	13
219	USA: Roanoke	311,993	65	42,757	8
220	JPN: Aomori	309,601	585	30,530	11
221	FR: Amiens	309,154	145	29,772	17
222	FR: Limoges	307,992	114	29,160	12
223	DE: Pforzheim	307,352	536	38,695	15
224	DE: Rosenheim	307,074	213	37,486	18
225	DE: Bremerhaven	307,055	149	30,604	11
226	ES: Oviedo	304,133	407	28,336	8
227	DE: Schwerin	303,031	64	29,287	11
228	DE: Paderborn	298,853	281	37,865	12
229	FR: Le Havre	297,916	537	33,328	16
230	ES: Gijon	296,163	863	25,587	9
231	UK: Lincoln	296,097	143	26,721	11
232	USA: Nashville	294,618	427	58,031	10
233	UK: Blackburn with Darwen	285,594	489	28,709	10
234	ES: Marbella	285,326	509	23,226	13
235	DE: Neubrandenburg	278,044	48	27,794	12
236	DE: Hildesheim	276,440	248	29,355	12
237	DE: Flensburg	274,656	138	33,897	9
238	DE: Reutlingen	273,578	272	42,986	16
239	DE: Kaiserslautern	273,554	226	30,696	14
240	FR: Dunkerque	273,513	425	30,390	17
241	FR: Annecy	272,588	256	32,023	15
242	FR: Besancon	270,164	138	29,345	15
243	DE: Schweinfurt	267,890	136	44,541	16
244	FR: Pau	267,702	146	34,751	11
245	FR: Poitiers	266,275	118	31,591	13
246	ES: Vitoria	264,719	200	46,600	10
247	DE: Gorlitz	264,402	130	27,186	17
248	UK: Dundee City	264,390	121	29,547	8
249	UK: Guildford	263,440	764	49,131	12
250	JPN: Obihiro	262,830	150	31,404	9
251	DE: Wetzlar	251,578	255	35,552	13
252	ES: Elche/Elx	249,200	4224	25,221	12
253	DE: Trier	248,567	227	33,037	13
**Bottom 127**	**USA: Tuscaloosa**	**244,054**	**43**	**43,490**	**11**
